# Dynamic Fracture Strength Prediction of HPFRC Using a Feature-Weighted Linear Ensemble Approach

**DOI:** 10.3390/ma18174097

**Published:** 2025-09-01

**Authors:** Xin Cai, Yunmin Wang, Yihan Zhao, Liye Chen, Jifeng Yuan

**Affiliations:** 1 Sinosteel Maanshan General Institute of Mining Research Co., Ltd., Maanshan 243000, China; xincai@csu.edu.cn (X.C.); maswym@126.com (Y.W.); 2 School of Resources and Safety Engineering, Central South University, Changsha 410083, China; cly991026@163.com; 3 PowerChina Zhongnan Engineering Co., Ltd., Changsha 410014, China; happy522923654@163.com

**Keywords:** high-performance fiber-reinforced concrete, dynamic fracture strength, strain rate dependency, machine learning, ensemble learning, model interpretability

## Abstract

Owing to its excellent crack resistance and durability, High-Performance Fiber-Reinforced Concrete (HPFRC) has been extensively applied in engineering structures exposed to extreme loading conditions. The Mode I dynamic fracture strength of HPFRC under high-strain-rate conditions exhibits significant strain-rate sensitivity and nonlinear response characteristics. However, existing experimental methods for strength measurement are limited by high costs and the absence of standardized testing protocols. Meanwhile, conventional data-driven models for strength prediction struggle to achieve both high-precision prediction and physical interpretability. To address this, this study introduces a dynamic fracture strength prediction method based on a feature-weighted linear ensemble (FWL) mechanism. A comprehensive database comprising 161 sets of high-strain-rate test data on HPFRC fracture strength was first constructed. Key modeling variables were then identified through correlation analysis and an error-driven feature selection approach. Subsequently, six representative machine learning models (KNN, RF, SVR, LGBM, XGBoost, MLPNN) were employed as base learners to construct two types of ensemble models, FWL and Voting, enabling a systematic comparison of their performance. Finally, the predictive mechanisms of the models were analyzed for interpretability at both global and local scales using SHAP (SHapley Additive exPlanations) and LIME (Local Interpretable Model-agnostic Explanations) methods. The results demonstrate that the FWL model achieved optimal predictive performance on the test set (R^2^ = 0.908, RMSE = 2.632), significantly outperforming both individual models and the conventional ensemble method. Interpretability analysis revealed that strain rate and fiber volume fraction are the primary factors influencing dynamic fracture strength, with strain rate demonstrating a highly nonlinear response mechanism across different ranges. The integrated prediction framework developed in this study offers the combined advantages of high accuracy, robustness, and interpretability, providing a novel and effective approach for predicting the fracture behavior of HPFRC under high-strain-rate conditions.

## 1. Introduction

High-Performance Fiber-Reinforced Concrete (HPFRC) is a composite material consisting of randomly dispersed short steel or synthetic fibers embedded within a high-strength cementitious matrix. It exhibits exceptional mechanical properties, including elevated strength, improved toughness, and superior crack control capabilities [1,2]. Owing to its outstanding energy dissipation capacity and crack-bridging effects, HPFRC has been widely adopted in critical infrastructure projects demanding high structural ductility and safety, such as bridges, tunnels, subway systems, high-speed railways, and blast/impact-resistant structures [3,4,5,6]. Under extreme loading conditions such as impacts, blasts, or seismic events, numerous materials are frequently subjected to high-strain-rate loading [7,8]. Their dynamic mechanical behavior exhibits significant deviations from static performance, particularly in the strain-rate-dependent fracture strength, which demonstrates pronounced rate dependency and a nonlinear response [9,10,11]. Therefore, establishing a predictive model that is high-accuracy, robust, and physically consistent is crucial for gaining deeper insights into the fracture behavior of HPFRC under dynamic loading and for advancing impact-resistant structural design.

Substantial experimental research has been conducted to investigate the mechanical response of HPFRC under dynamic loads. For instance, Tran and Kim [12] employed a Strain Energy Frame Impact Machine (SEFIM) to measure direct tensile properties at strain rates up to 92 s^−1^. Wahba and Marzouk [13] utilized Fiber Bragg Grating sensors to monitor dynamic stress–strain responses in large-scale HPFRC specimens (up to 1 m in length). Furthermore, Dang and Kim [14] systematically evaluated the fracture behavior of nanoparticle-reinforced Ultra-High-Performance Fiber-Reinforced Concrete (UHPFRC) across strain rates ranging from 0.0003 to 156 s^−1^. Their findings confirmed the high-strain-rate sensitivity of fracture strength, which is significantly influenced by fiber volume fraction and fiber morphology.

However, despite significant advances in experimental methods for characterizing the fracture behavior of High-Performance Fiber-Reinforced Concrete (HPFRC) under high strain rates, several challenges remain, including the high cost of equipment, difficulties in standardizing loading protocols, and limited data acquisition capabilities. These challenges not only hinder systematic modeling of the material’s true response under extreme conditions but also impede in-depth analysis of the multivariate coupling mechanisms governing fracture behavior. To address these gaps, researchers have increasingly adopted data-driven modeling approaches, leveraging machine learning techniques to capture the nonlinear behavior of HPFRC under complex loading conditions [15,16,17,18,19]. For instance, Su et al. [20] employed ensemble learning methods to predict the compressive strength, splitting tensile strength, and flexural strength of fiber-reinforced concrete (FRC). The results indicated that the GBDT regression model performed best in predicting compressive and flexural strength, while the XGBOOST regression model showed the highest accuracy in predicting splitting tensile strength. Nguyen et al. [15] developed a multi-model predictive framework for HPFRC fracture strength under high strain rates using Random Forest (RF), Extreme Gradient Boosting (XGBoost), and Deep Neural Networks (DNNs). They introduced a hybrid human–machine evaluation mechanism to enhance model generalizability and reliability under small-sample constraints. Their results demonstrated the superior predictive accuracy and stability of the XGBoost model, validating its efficacy for strain-rate-sensitive problems. Progress has also been made in data-driven modeling for static or quasi-static conditions. Zheng et al. [17] compiled a flexural strength database for Steel Fiber-Reinforced Concrete (SFRC) and employed three ensemble learning models—Gradient Boosting (GB), RF, and XGBoost—to predict 28-day strength. The GB model achieved the highest accuracy (R^2^ = 0.96), highlighting the potential of ensemble algorithms for the rapid mechanical performance assessment of SFRC. Abuodeh et al. [19] focused on compressive strength prediction of Ultra-High-Performance Concrete (UHPC), utilizing an Artificial Neural Network (ANN) combined with Sequential Feature Selection (SFS) and Neural Interpretation Diagram (NID) methods to identify key material components. This approach improved both model accuracy and interpretability, offering valuable insights into explaining “black-box” models. Nevertheless, current studies have primarily focused on static or quasi-static loading conditions, leaving the accurate prediction of HPFRC fracture strength under high-strain-rate scenarios insufficiently addressed. While some recent works have explored interpretable ensemble modeling approaches, their use of fixed weighting strategies limits adaptability to varying input conditions and reduces predictive accuracy in complex, nonlinear high-strain-rate environments. A high-precision dynamic response modeling framework remains lacking. Furthermore, existing studies have yet to fully elucidate the dynamic interactions and regulatory mechanisms among key variables during fracture strength prediction.

To overcome the aforementioned limitations, this study proposes a feature-weighted linear ensemble (FWL) approach for high-precision prediction of the Mode I dynamic fracture strength of HPFRC under complex nonlinear conditions. The proposed method integrates six heterogeneous base learners—KNN, RF, SVR, LGBM, XGBoost, and MLPNN—forming a structurally diverse and functionally complementary model ensemble. This configuration not only preserves the local fitting capacity of individual models but also enhances the generalization performance of the overall framework. Distinct from conventional ensemble methods, FWL incorporates a feature-driven dynamic weighting mechanism at the ensemble layer, enabling the model to adaptively adjust fusion weights in real time based on the input conditions. This mechanism significantly improves the model’s adaptability to local heterogeneity and nonlinear variations in the input space, thereby enhancing its robustness and predictive accuracy under high-strain-rate loading scenarios. Moreover, to enhance interpretability and provide insight into the internal decision-making process of the model, this study incorporates two model-agnostic explanation techniques: SHapley Additive Explanations (SHAP) and Local Interpretable Model-agnostic Explanations (LIME). These techniques are employed to analyze the model’s behavior from both global feature importance and local inference perspectives. By systematically examining SHAP dependence relationships and local interpretation results across different strain-rate intervals, this study identifies the key features and nonlinear interactions that govern the dynamic evolution of HPFRC fracture strength. This offers valuable data-driven support for constitutive modeling and mechanistic understanding of material fracture behavior.

## 2. Dataset Construction and Feature Engineering

### 2.1. Dataset Construction and Analysis

The dataset employed in this study was compiled from published experimental investigations [1,2,11,12]. These studies systematically investigated the dynamic tensile behavior of High-Performance Fiber-Reinforced Concrete (HPFRC), with specific attention to Mode I dynamic fracture strength and associated fracture characteristics, over a strain-rate range from 10^−4^ s^−1^ to 10^2^ s^−1^. Experimental methodologies encompassed direct tensile tests, drop-weight impact tests, and specialized high-strain-rate loading apparatuses (e.g., SEFIM system). Data encompassing material strength, fiber geometric parameters, loading rates, and other dimensions exhibit high physical relevance and representativeness. Due to inconsistent reporting across source studies, some variables have fewer samples, which may introduce minor sampling bias. However, there are limited missing data and key features are well-represented. Meanwhile, during dataset assembly, duplicate and anomalous samples were rigorously excluded, while multiple test results under identical mix proportions were retained to authentically capture experimental variability under dynamic loading conditions. The finalized dataset comprises 161 samples.

Table 1 provides a comprehensive description of all input and output variables incorporated within the dataset. Eight input variables were considered, encompassing mortar compressive strength, specimen cross-sectional area, fiber diameter, fiber shape, fiber length, fiber volume fraction, fiber tensile strength, and strain rate. These variables represent key controlling factors with potential influence on the dynamic mechanical behavior of HPFRC. Notably, strain rate, serving as the primary parameter reflecting loading rate, exhibits an exceptionally broad range (0.000167 s^−1^ to 100 s^−1^). This range comprehensively spans loading states from quasi-static to highly dynamic impact conditions, thereby endowing the predictive model with enhanced adaptability and generalization capability. The output variable is fracture strength under various loading conditions, ranging from 6.0 to 45.0 MPa with a mean value of 20.66 MPa. This range reflects the dynamic response amplitude of the material across different strain rates. Analysis of the input features suggests that variations in fracture strength are not only governed by the inherent mechanical properties of the concrete matrix but are also critically dependent on the complex coupling effects between fiber characteristics and loading rate.

The statistical characteristics presented in Table 1 reveal distinct distribution patterns across most variables. For instance, mortar compressive strength ranges from 56 to 180 MPa, with a mean of 152.62 MPa, indicating that the selected concrete systems predominantly belong to high-strength grades. Regarding fiber parameters, fiber length varies between 13 and 30 mm, and multiple shapes are included (hooked, twisted, long smooth, short smooth, hybrid), demonstrating diversity in the fiber reinforcement mechanisms under investigation. The database constructed in this study exhibits strong representativeness and completeness in terms of sample size, variable dimensionality, and distribution coverage. Moreover, it effectively captures the physical relationships between high-strain-rate loading mechanisms, which are central to this research, and fiber reinforcement parameters. This solid foundation provides reliable support for the subsequent predictive modeling.

### 2.2. Feature Engineering and Preprocessing

**Figure 1:**
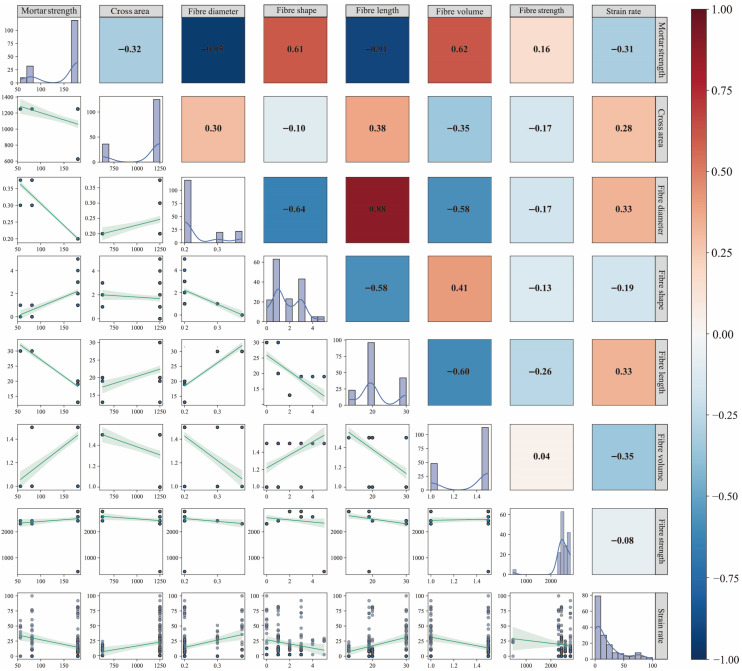
Spearman correlation matrix and joint distribution plot of input variables.

To enhance the stability and generalizability of predictive models, feature selection analysis was conducted on the established database, considering both linear correlations among variables and their actual contributions to predictive performance. As illustrated in Figure 1, the Spearman correlation heatmap and joint distribution plot reveal strong correlations between certain input variables, indicating potential multicollinearity risks. Notably, significant negative correlations were observed between fiber diameter and fiber length (Corr = −0.91) and between fiber diameter and mortar strength (ρ = −0.95). These correlations suggest covariation in experimental design parameters, which may lead to parameter instability if directly used in regression modeling.

**Figure 2:**
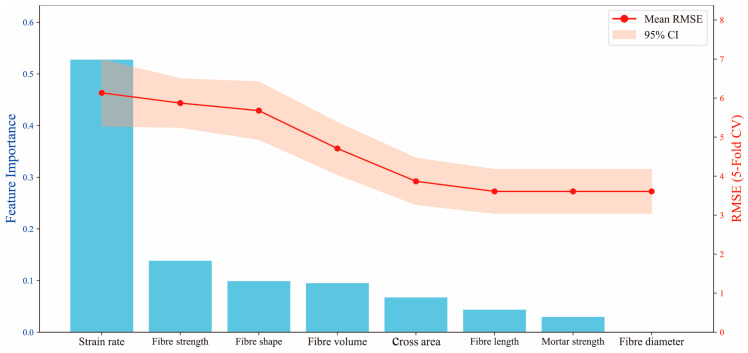
Feature importance and cross-validation RMSE evolution based on stepwise feature addition.

To address the above issues, this study employs a hybrid feature selection methodology integrating feature importance ranking with a stepwise introduction strategy. An initial model is first trained using all features to extract the importance ranking of input variables. Then, variables are sequentially introduced in descending order of importance. For each feature subset, the Root-Mean-Squared Error (RMSE) and its 95% confidence interval are assessed via five-fold cross-validation. The cross-validation results were monitored not only for minimizing error but also for ensuring stability across folds, thereby supporting robust feature selection. Analysis of the error evolution trends enables identification and elimination of redundant or interfering features, ultimately yielding a streamlined and efficient feature set. Figure 2 presents the resulting error evolution profile. Strain rate emerges as the most predictive single variable, achieving a low RMSE even under single-feature input conditions. Its significance substantially surpasses that of other variables, underscoring its dominant role in governing fracture strength responses under high-strain-rate loading. Fiber tensile strength and fiber shape rank as the subsequent influential factors, reflecting the impact of fiber reinforcement mechanisms on material failure processes under dynamic conditions. Notably, mortar compressive strength and fiber diameter not only exhibit strong correlations with other variables (Figure 1) but also demonstrate low feature importance (Figure 2). Their inclusion yields negligible positive gains in overall model performance, even inducing minor RMSE fluctuations. Consequently, these two variables are identified as redundant features and subsequently excluded from further analysis to streamline model architecture, mitigate overfitting risks, and enhance modeling efficiency and robustness.

**Figure 3:**
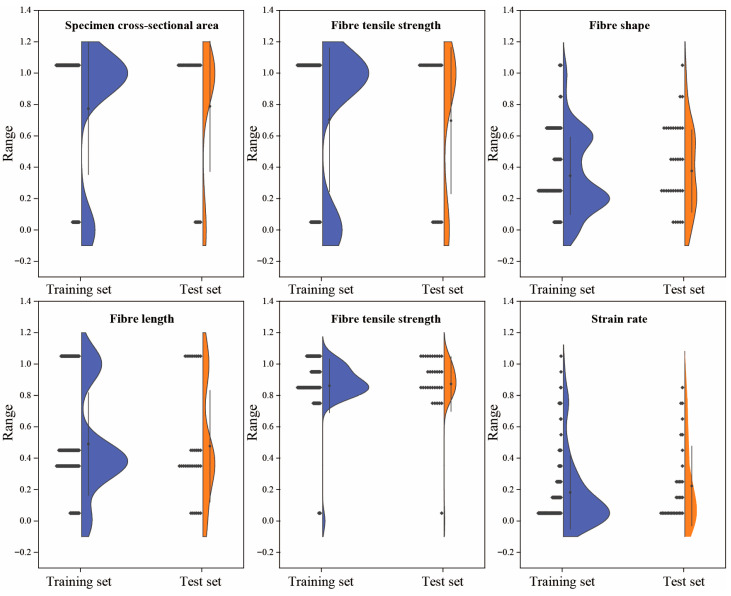
Distribution comparison of normalized input features between training and test sets.

To mitigate potential adverse effects of variable scale discrepancies on model convergence speed and prediction accuracy, all input variables underwent standardization prior to modeling. Furthermore, the dataset was randomly partitioned into a training set (128 samples) and a test set (33 samples) at an 8:2 ratio, enabling robust evaluation of model generalizability on unseen data. Figure 3 presents a comparative distribution analysis of normalized input features across training and test sets. The distributions are closely aligned across all variables, with no significant shifts or imbalances, indicating that the data partitioning strategy effectively preserves the original data structure. Standardization and data partitioning collectively establish optimal preconditions for subsequent model development, ensuring both numerical uniformity among features and statistical representativeness throughout the training–testing evaluation process.

## 3. Methods

### 3.1. Feature Weight Linear Stacking

**Figure 4:**
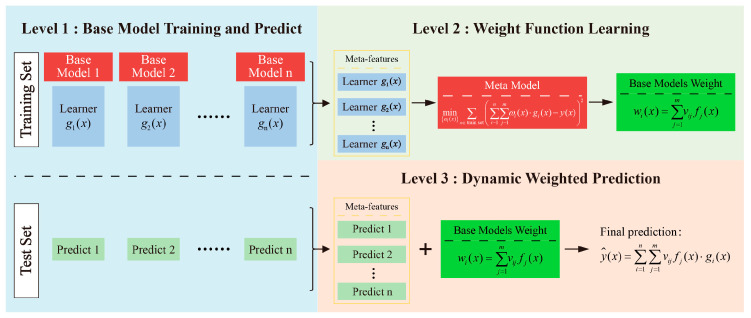
Framework of the feature-weighted learning (FWL) ensemble model.

Feature-weighted linear stacking is an ensemble learning approach that integrates the predictions of multiple base models through a dynamic weighting mechanism, allowing for non-uniform fusion of model outputs. The overall architecture can be divided into three hierarchical levels, as illustrated in Figure 4 [21]: base model learning, weight function learning, and dynamically weighted prediction. The training process comprises three main steps:

(1) Base model training and prediction generation: Initially, multiple heterogeneous or homogeneous base learners are trained on the training dataset. Each base model receives the original input features and outputs its preliminary prediction of the target variable. Upon completion of this stage, the predictions from all base models for each training sample are collected, forming a new set of inputs—referred to as meta-features—for the subsequent weight function learning.
(2) Weight function learning: In the second stage, a meta-model is constructed using the meta-features generated in the previous step to learn the relative credibility or contribution of each base model under varying input conditions. The objective is to minimize the weighted squared residual loss function:

(1) $$\underset{\left\{\omega_{i}(x)\right\}}{\min}\sum_{x\in \;\mathrm{train}\;\mathrm{set}}{\left(\overset{n}{\underset{i=1}{\sum }}\overset{m}{\underset{j=1}{\sum }}\omega_{i}(x)\cdot g_{i}(x)-y(x)\right)}^{2}$$

where $\omega_{i}(x)$ represents the prediction weight of the *j*-th base model under the input condition x, which must satisfy the normalization constraint:

(2) $$\sum_{j=1}^{n}\omega_{i}(x)=1,\;\omega_{i}(x)\geq 0$$

This optimization problem can be addressed by fitting a meta-model to learn the weight functions, thereby obtaining a dynamic weight distribution conditioned on the input features. The weight function is defined as

(3) $$w_{i}(x)=\overset{m}{\underset{j=1}{\sum }}v_{ij}f_{j}(x)$$

where $f_{j}(x)$ represents the raw weight primitive output by the family of weight-generating functions, m is the number of base models, and $v_{ij}$ denotes the learned adaptive weight coefficient for the *j*-th base model.

This mechanism allows input-dependent, localized adjustment of prediction weights by capturing the dynamic response behavior of each base model, thereby enhancing the overall nonlinear representation capacity and generalization performance of the ensemble.

(3) Dynamic Weighted Ensemble Prediction. In the prediction phase, once a test sample is input, each base model independently generates its prediction. These individual outputs are then aggregated using the dynamic weight functions $\omega_{i}(x)$ learned in the second stage, resulting in the final ensemble prediction:

(4) $$\hat{y}(x)=\overset{n}{\underset{i=1}{\sum }}\overset{m}{\underset{j=1}{\sum }}v_{ij}f_{j}(x)\cdot g_{i}(x)$$

Compared with traditional static-weighted ensemble methods, the FWL strategy explicitly models the prediction weights as functions of the input features, thereby significantly enhancing the model’s adaptability in nonlinear feature spaces.

### 3.2. Voting Regression

**Figure 5:**
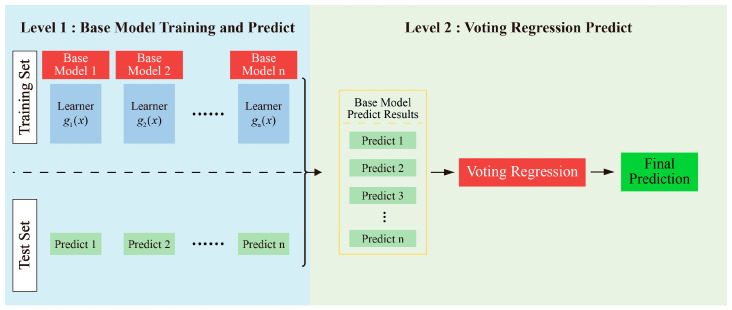
Framework of the Voting Regression ensemble method.

This study adopts Voting Regression as a benchmark method for performance comparison. Voting Regression integrates the predictive outputs of multiple base learners to enhance the overall stability and generalization capability of the model. Its fundamental architecture, as illustrated in Figure 5, consists of two main stages: base model training and ensemble prediction through voting.

(1) Base Model Training and Prediction (Stage I): Multiple independent regression models are constructed using the training dataset. Each model learns its individual regression mapping function based on the training data. Once trained, these base models generate independent prediction outputs on the test set as follows:

(5) $$\hat{y}(x)=g_{j}(x),j=1,2,\ldots ,n$$

(2) Voting Regression Prediction (Stage II): The individual predictions from all base models are aggregated to generate the final output. The core idea of the Voting Regression strategy is to compute the average of all base model predictions:

(6) $$\hat{y}(x)=\frac{1}{n}\sum_{j=1}^{n}{\hat{y}}_{j}(x)$$

Compared with a single model, the Voting ensemble can effectively mitigate the influence of individual model errors on the overall performance. It offers robust generalization capability and is straightforward to implement.

### 3.3. Machine Learning Models

To construct an ensemble learning framework with high diversity and complementary capabilities, this study selects six representative machine learning regression models as base learners: the instance-based algorithm (K-Nearest Neighbors, KNN), ensemble tree models (Random Forest, RF; Light Gradient Boosting Machine, LGBM; and Extreme Gradient Boosting, XGBoost), kernel-based method (Support Vector Regression, SVR), and a feedforward neural network (Multi-Layer Perceptron Neural Network, MLPNN). All machine learning models were implemented in Python (version 3.9). The Random Forest and Support Vector Regression were executed using the scikit-learn package (version 1.0.2). The Extreme Gradient Boosting (XGBoost) model was implemented using the xgboost library (version 1.6.2), and the Light Gradient Boosting Machine (LightGBM) using the lightgbm library (version 3.3.5). The Multi-Layer Perceptron Neural Network was built with the scikit-learn MLPRegressor module. These models encompass a range of mainstream modeling paradigms and exhibit distinct structural mechanisms and fitting capacities, allowing a comprehensive comparison of different strategies in the context of predicting the dynamic fracture strength of High-Performance Fiber-Reinforced Concrete (HPFRC). The fundamental principles of each base model are briefly introduced in this section, and detailed algorithmic descriptions can be found in the relevant literature.

#### 3.3.1. K-Nearest Neighbors (KNN)

K-Nearest Neighbors (KNN) is a non-parametric, instance-based regression method that operates under the principle that “samples with similar features tend to exhibit similar responses.” Unlike parametric models, KNN requires no explicit training process, making it conceptually simple and particularly well-suited for small datasets with well-defined local structures. To predict the target value of a new input instance, the KNN algorithm computes the distance between the input and all samples in the training set—commonly using the Euclidean distance metric [22]:

(7) $$d\left(x,x_{j}\right)=\sqrt{\overset{n}{\underset{k=1}{\sum }}{\left(x_{k}-x_{jk}\right)}^{2}}$$

where x denotes the input sample to be predicted, $x_{j}$ represents the *j*-th training sample, and n is the number of input features, with $x_{k}$ and $x_{jk}$ denoting the values of the *k*-th feature for the input and training sample, respectively. The model selects the *K*-Nearest Neighbors with the smallest distances to x, and computes the predicted output as the average of their corresponding target values $y_{i}$:

(8) $$\hat{y}=\frac{1}{K}\sum_{j=1}^{K}y_{i}$$

#### 3.3.2. Random Forest (RF)

Random Forest is an ensemble learning algorithm based on the Bagging (Bootstrap Aggregating) principle, consisting of a collection of regression trees. Each individual tree is trained on a bootstrap sample drawn with replacement from the original dataset. The core idea of Random Forest is to reduce the risk of overfitting associated with single models by aggregating the predictions from multiple weak learners—akin to taking the “average opinion” of a crowd. During training, each tree is built using a subset of samples, and at each split node, a random subset of features is considered for determining the optimal split. This “double randomness” in both samples and features enhances model diversity and robustness. For a given test input *x*, the final prediction is computed as the average of the outputs from all regression trees [23]:

(9) $$\hat{y}=\frac{1}{T}\sum_{t=1}^{T}f_{t}(x)$$

where T denotes the total number of trees in the forest, and $f_{t}(x)$ represents the output of the *t*-th regression tree. Random Forest is well-suited for handling nonlinear and high-dimensional data due to its strong robustness and inherent resistance to overfitting. By aggregating diverse tree-based predictions, it achieves stable performance even under complex feature interactions and noisy input conditions.

#### 3.3.3. Support Vector Regression (SVR)

Support Vector Regression (SVR) is an extension of the Support Vector Machine (SVM) for regression tasks. Its core idea is to construct an optimal regression function in a high-dimensional feature space such that the prediction error falls within a specified tolerance *ε*, while maintaining strong generalization capability. The SVR function is typically expressed as [24]

(10) $$\hat{y}(x)=\langle \omega ,\phi (x)\rangle +b$$

where $\hat{y}(x)$ denotes the predicted value of the input sample, ϕ(x) is a nonlinear mapping from the input space to a higher-dimensional feature space, and ω and b are the weight vector and bias term, respectively. SVR is particularly effective in solving nonlinear, high-dimensional, and small-sample regression problems, owing to its capacity to control model complexity and prevent overfitting via margin maximization.

#### 3.3.4. Light Gradient Boosting Machine (LightGBM)

LightGBM is an optimized implementation of the Gradient Boosting Decision Tree (GBDT) algorithm, specifically designed to improve training speed and memory efficiency, making it particularly well-suited for large-scale and high-dimensional sparse datasets. While retaining the fundamental structure of GBDT, LightGBM enhances performance through three key techniques: (1) histogram-based feature splitting, which discretizes continuous features into discrete bins, thereby reducing computational complexity; (2) leaf-wise growth strategy, which prioritizes expanding the leaf node that yields the greatest loss reduction rather than growing the tree level-wise; (3) accelerated mechanisms, such as Gradient-based One-Side Sampling (GOSS) and Exclusive Feature Bundling (EFB), which reduce the sample size and feature dimensionality without significant loss in accuracy. The model formulation is consistent with traditional GBDT approaches [25]:

(11) $$\hat{y_{i}}=\sum_{m=1}^{M}\eta \cdot f_{m}(x_{i})$$

where $f_{m}$ denotes the prediction of the *m*-th decision tree.

#### 3.3.5. Extreme Gradient Boosting (XGBoost)

XGBoost is a highly efficient variant of the Gradient Boosting Decision Tree (GBDT) framework. Compared to traditional gradient boosting methods, it delivers significant improvements in training speed, model accuracy, and generalization capability. A key innovation of XGBoost lies in its use of second-order gradient information (i.e., both the first and second derivatives) to improve the precision of gradient estimation. In addition, it incorporates a regularization term into the objective function, which penalizes model complexity to mitigate overfitting and enhance robustness. The objective function of XGBoost is defined as [26]

(12) $$L^{\left(t\right)}=\sum_{i=1}^{n}l(y_{i},{\hat{y_{i}}}^{\left(t\right)})+\Omega (f_{t})$$

(13) $$\Omega (f_{t})=\gamma T+\frac{1}{2}\lambda {\left\|\omega \right\|}^{2}$$

where *n* is the number of samples; $l(y_{i},{\hat{y_{i}}}^{\left(t\right)})$ is the loss function; $y_{i}$ represents the true value of the *i*-th sample; ${\hat{y_{i}}}^{\left(t\right)}$ is the predicted value of the *i*-th sample after the first t decision trees are combined; $\Omega (f_{t})$ is the regularization term, introduced to control model complexity and prevent overfitting; γ is the shrinkage coefficient; T is the number of leaf nodes; λ is the regularization parameter; and ω denotes the weight of the leaf nodes.

#### 3.3.6. Multi-Layer Perceptron Neural Network (MLPNN)

The Multi-Layer Perceptron Neural Network (MLPNN) is a structurally simple yet powerful feedforward neural network capable of approximating any continuous function. The network architecture consists of an input layer, one or more hidden layers, and an output layer, with information propagating forward through the layers in sequence. Each layer performs a linear transformation followed by a nonlinear activation function, as expressed by [27]

(14) $$a^{\left(l\right)}=\sigma (W^{\left(l\right)}a^{\left(l-1\right)}+b^{\left(l\right)})$$

where $a^{\left(l\right)}$ is the output of the *l*-th layer; $W^{\left(l\right)}$ and $b^{\left(l\right)}$ are the weight matrix and bias vector, respectively; and σ(⋅) represents the activation function (e.g., ReLU or Tanh). For regression tasks, MLPNN typically adopts the Mean Squared Error (MSE) as its loss function:

(15) $$MSE=\frac{1}{n}{\sum_{i=1}^{n}\left(y_{i}-\hat{y_{i}}\right)}^{2}$$

where $y_{i}$ and $\hat{y_{i}}$ represent the actual and predicted values, respectively, and n is the number of samples.

The model is trained using the backpropagation algorithm in conjunction with optimization algorithms such as Adam to update the network parameters. Due to its capacity to capture complex nonlinear relationships, MLPNN is particularly well-suited for modeling intricate input–output mappings in regression problems.

### 3.4. Prediction Framework Construction

**Figure 6:**
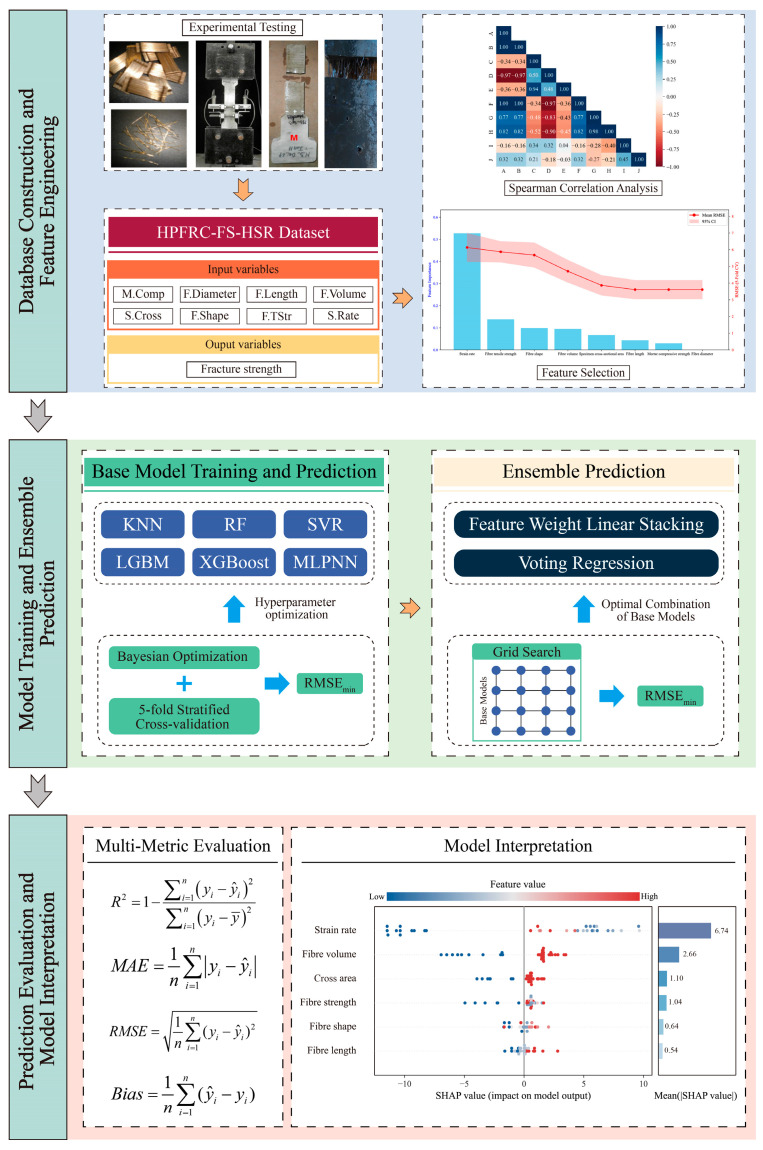
Overall workflow of the proposed HPFRC fracture strength prediction framework.

To systematically predict the fracture strength of high-performance fiber-reinforced concrete (HPFRC) under high-strain-rate conditions, this study develops a comprehensive predictive framework encompassing data preprocessing, base model training, ensemble integration, and model interpretation. As illustrated in Figure 6, the overall workflow is structured into three core modules: data processing and feature engineering, model training and ensemble prediction, and model evaluation and interpretation.

(1) Data Processing and Feature Engineering

A fracture strength dataset (HPFRC-FS-HSR Dataset) was established using experimental data collected from previously published literature. The dataset includes eight input variables: fiber diameter, length, volume fraction, tensile strength, shape, specimen size, mortar compressive strength, and applied strain rate. The output variable is fracture strength. To enhance feature representation quality, a combination of Spearman correlation analysis and error-based stepwise feature selection was employed to identify and eliminate redundant variables. Only the most relevant features were retained for model development.

(2) Model Training and Ensemble Prediction

Six representative regression models—KNN, RF, SVR, LGBM, XGBoost, and MLPNN—were selected as base learners. Their hyperparameters were optimized using Bayesian optimization in conjunction with five-fold cross-validation. Subsequently, two ensemble strategies were constructed to enhance overall predictive performance. The optimal combination of base models was determined via grid search, ensuring sufficient model diversity and complementary performance within the ensemble structure.

(3) Model Evaluation and Interpretation

The final predictive models were evaluated using multiple performance metrics, including the coefficient of determination (R^2^), mean absolute error (MAE), root-mean-square error (RMSE), and bias, to comprehensively assess model accuracy, stability, and generalization. Furthermore, SHapley Additive Explanations (SHAP) and Local Interpretable Model-agnostic Explanations (LIME) were introduced to quantify the marginal contributions of input variables to model predictions. These interpretability tools provide insight into the decision-making logic of the models and identify key influencing factors, thereby supporting physically meaningful and traceable modeling of mechanical responses.

## 4. Performance Evaluation Methods

To comprehensively evaluate the performance of the proposed regression models in predicting the fracture strength of High-Performance Fiber-Reinforced Concrete (HPFRC), this study establishes a systematic evaluation framework that integrates multiple error metrics and model interpretability techniques. For predictive accuracy assessment, four representative regression indicators—coefficient of determination (R^2^), mean absolute error (MAE), root-mean-square error (RMSE), and bias—are employed. In terms of model interpretability, two widely adopted explainability approaches, i.e., SHapley Additive Explanations (SHAP) and Local Interpretable Model-agnostic Explanations (LIME), are introduced to elucidate the influence mechanisms of input features on model outputs.

### 4.1. Evaluation Metric

The performance of regression models is typically assessed by quantifying the discrepancy between predicted and actual values. In this study, four commonly used evaluation metrics are employed:

(1) Coefficient of Determination (*R^2^*): This metric measures the proportion of variance in the target variable that is explained by the model. It is defined as [28]

(16) $$R^{2}=1-\frac{\sum_{i=1}^{n}{\left(y_{i}-\hat{y_{i}}\right)}^{2}}{\sum_{i=1}^{n}{\left(y_{i}-\bar{y}\right)}^{2}}$$

where $\bar{y}$ denotes the mean of actual values. An $R^{2}$ value closer to 1 indicates better predictive performance.

(2) Mean Absolute Error (*MAE*): *MAE* quantifies the average absolute difference between predicted and actual values, providing a measure that is less sensitive to outliers. It is calculated as [29]

(17) $$MAE=\frac{1}{n}\sum_{i=1}^{n}\left|y_{i}-{\hat{y}}_{i}\right|$$

A lower *MAE* indicates that the model’s predictions are, on average, closer to the true values, making it suitable for evaluating general predictive stability.

(3) Root-Mean-Square Error (*RMSE*): *RMSE* evaluates the square root of the average squared differences between predicted and actual values, making it more sensitive to large errors [28]:

(18) $$RMSE=\sqrt{\frac{1}{n}\sum_{i=1}^{n}{\left(y_{i}-\hat{y_{i}}\right)}^{2}}$$

This metric penalizes large deviations more heavily, thus providing insight into the model’s ability to avoid significant prediction errors.

(4) Systematic Bias (*Bias*): *Bias* quantifies the average directional deviation between predictions and actual values, capturing whether the model systematically overestimates or underestimates the target [29]:

(19) $$Bias=\frac{1}{n}\sum_{i=1}^{n}\left({\hat{y}}_{i}-y_{i}\right)$$

A *Bias* close to 0 indicates that the model is generally unbiased, while positive or negative values suggest a tendency toward consistent overprediction or underprediction, respectively. By jointly applying these four metrics (*R^2^*, *MAE*, *RMSE*, and *Bias*), a comprehensive assessment of model performance is achieved, ensuring the reliability and robustness of the predictive framework under practical engineering conditions.

### 4.2. Shapley Additive Explanations (SHAP)

SHAP (SHapley Additive exPlanations) is a unified model interpretation method rooted in game theory that enables both global and local explanations by quantitatively evaluating the contribution of each input feature to an individual prediction. The core concept of SHAP is based on the Shapley value, which ensures a fair allocation of contributions among features by averaging their marginal effects across all possible feature subsets. For a given input instance *x*, SHAP expresses the model output as [30]

(20) $$f(x)=\phi_{0}+\sum_{j=1}^{M}\phi_{j}$$

where f(x) denotes the model’s prediction for sample x, $\phi_{0}$ is the baseline value (i.e., the expected prediction when all features are missing), and $\phi_{j}$ represents the marginal contribution of feature j to the current prediction. Each $\phi_{j}$ is computed using the Shapley value formula:

(21) $$\phi_{j}=\sum_{S\subseteq F\backslash \left\{j\right\}}\frac{\left|S\right|!(\left|F\right|-\left|S\right|-1)!}{\left|F\right|!}\left[f_{S\cup \left\{j\right\}}(x_{S\cup \left\{j\right\}})-f_{S}(x_{S})\right]$$

where F is the full set of input features, S denotes a subset of excluded features, and $f_{S}$ is the model’s prediction based only on the feature subset S. The term inside the brackets captures the marginal improvement in prediction when feature j is added to subset S. By averaging over all permutations, SHAP ensures that feature importance rankings are both fair and consistent.

### 4.3. Local Interpretable Model-Agnostic Explanations (LIME)

LIME (Local Interpretable Model-agnostic Explanations) is a local, model-agnostic interpretation technique that approximates the prediction behavior of complex models using simple, interpretable surrogate models—such as linear regression—within a local neighborhood of the instance being explained. The core idea is to generate a faithful local approximation that reflects how the original model behaves in the vicinity of a specific input sample.

In practice, LIME generates a set of perturbed samples $x^{\prime }$ around the original instance x, obtains their corresponding predictions from the black-box model, and assigns weights to each perturbed sample based on proximity (e.g., using a Gaussian kernel on Euclidean distances). It then fits an interpretable model g by minimizing the following objective [31]:

(22) $$\mathrm{explanation}(x)=\arg\underset{g\in G}{\min}L\left(f,g,\pi_{x}\right)+\Omega (g)$$

where $L\left(f,g,\pi_{x}\right)$ denotes the weighted loss between the predictions of the complex model f and the surrogate model g, $\pi_{x}$ is the proximity-based weighting function, and Ω(g) is a regularization term that penalizes the complexity of the interpretable model. LIME is particularly effective for tracing the decision-making logic of black-box models—such as neural networks or ensemble learners—offering intuitive and actionable insights into local feature contributions.

## 5. Results and Discussion

### 5.1. Comparative Analysis of Model Performance

**Figure 7:**
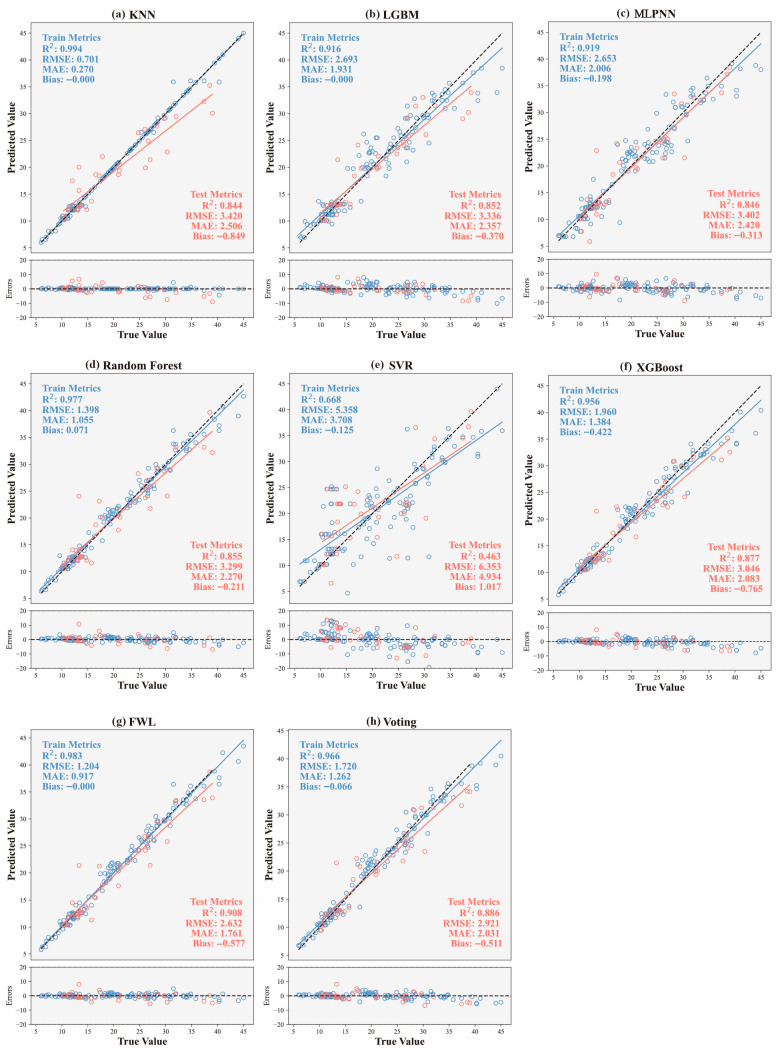
Comparison of predicted versus true values and residual errors for all models on training and test datasets: (**a**) KNN; (**b**) LGBM; (**c**) MLPNN; (**d**) RF; (**e**) SVR; (**f**) XGBoost; (**g**) FWL; (**h**) Voting.

In this section, we compare the fracture strength prediction performance of six individual base learners (KNN, RF, SVR, LGBM, XGBoost, and MLPNN) as well as two ensemble models: the proposed feature-weighted linear (FWL) ensemble and the conventional Voting Regression method. Figure 7 illustrates the predictive performance of all models on training and test datasets, including predicted-versus-true value distributions and error plots, supplemented by multidimensional evaluation metrics (R^2^, RMSE, MAE, Bias). Comparative results reveal that while most base models exhibit strong fitting capabilities on the training set (R^2^ > 0.90), their performance diverges significantly on the test set, reflecting inherent differences in generalization ability. For instance, RF and XGBoost achieve relatively high test-set R^2^ values (0.855 and 0.877, respectively), with well-controlled errors, demonstrating robust nonlinear modeling capabilities. In contrast, SVR substantially underperforms (R^2^ = 0.463, RMSE = 6.353), exhibiting a pronounced positive bias (+1.017). This indicates its limited robustness to data distribution under high-strain-rate conditions and susceptibility to local anomalies.

Regarding the ensemble models, both Voting and FWL demonstrated superior overall performance compared to all individual models, fully validating the effectiveness of ensemble strategies in enhancing model stability and prediction accuracy. The FWL model achieved globally optimal predictive performance on the test set, with an R^2^ value of 0.908, RMSE of 2.632, MAE of only 1.761, and a Bias close to zero (−0.577). These metrics indicate the absence of significant systematic bias in the predictions, with errors demonstrating uniform convergence characteristics. Compared to the Voting model (R^2^ = 0.886, RMSE = 2.921), FWL exhibited a marked improvement in accuracy, highlighting the significant efficacy of its introduced feature-dependent weighting mechanism. Specifically, FWL incorporates the original input features into the meta-model layer as inputs to the weighting function. This enables input-responsive dynamic adjustment of the model fusion weights, thereby effectively capturing the nonlinear evolution patterns of fracture strength under varying combinations of strain rates and fiber parameters. This modeling strategy overcomes the limitations of fixed weighting inherent in traditional ensemble methods, exhibiting stronger adaptability when handling data structures characterized by significant nonlinearity and local heterogeneity.

Further observation of the error distribution plot below Figure 7 reveals that FWL consistently maintains smaller deviations and reduced error fluctuations across different fracture strength intervals. Prediction points are also highly concentrated near the diagonal line. This error distribution pattern signifies the model’s capability to accurately capture both global and local data characteristics, particularly demonstrating excellent robustness and generalization ability within the medium-to-high strength range. This capability holds significant value for engineering safety assessments.

In conclusion, the FWL model exhibited exceptional predictive accuracy and stability on the dataset constructed in this study, outperforming not only all individual base learners but also significantly surpassing the traditional Voting ensemble method. Its core advantage lies in the dynamic weighting mechanism driven by the original input features. This mechanism allows the model to flexibly adapt to complex response patterns under diverse input conditions, providing a more refined and scalable modeling approach for the intelligent prediction of HPFRC dynamic fracture behavior under high strain rates.

### 5.2. Model Interpretation

#### 5.2.1. Multi-Level SHAP Interpretation of the FWL Model

SHapley Additive exPlanations (SHAP) analysis was conducted to elucidate the underlying mechanisms of the proposed FWL ensemble model for predicting the dynamic fracture strength of HPFRC under high-strain-rate conditions. Given the multi-learner architecture of the FWL model, interpretability analysis was performed at two levels: the base-learner layer and the ensemble-output layer. This systematic approach reveals the marginal contributions of input variables to prediction outcomes and their response patterns.

**Figure 8:**
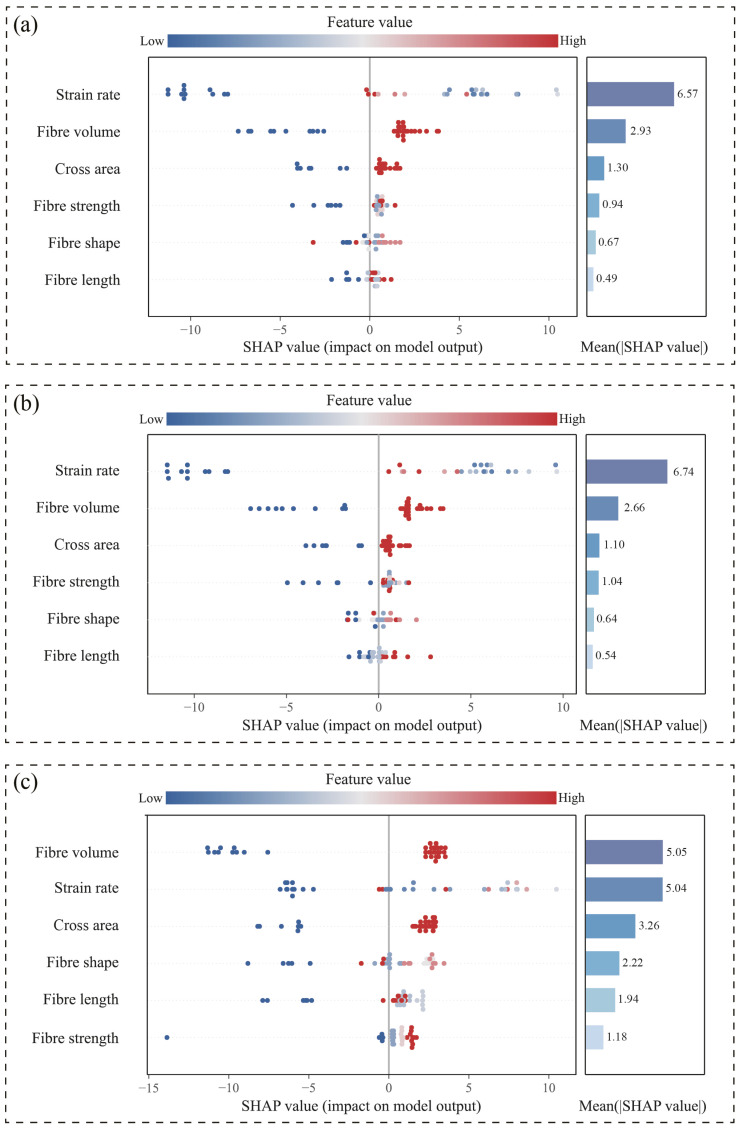
Combined SHAP beeswarm and mean importance plots of base learners: (**a**) XGBoost, (**b**) Random Forest, and (**c**) SVR.

Figure 8 presents integrated SHAP visualizations for the three top-performing base learners (XGBoost, RF, and SVR) within the FWL framework, combining beeswarm plots with mean absolute SHAP value bar charts. The beeswarm plots illustrate the directional impact (positive/negative) and distribution characteristics of input variables across samples, while the bar charts quantify global feature importance. Key findings indicate the following:

(1) Strain rate consistently dominates across all base learners, particularly in the RF model (mean SHAP value: 6.74), confirming its role as the primary predictor.
(2) Fiber volume and cross-sectional area subsequently ranked highest in importance, demonstrating stable significance and highlighting the marked dependence of fracture strength predictions on structural parameters (e.g., loading rate and fiber configuration).
(3) SVR exhibits a narrower dispersion of SHAP values with lower magnitudes, indicating limited responsiveness to sample variations and an inability to capture nonlinear relationships in fracture strength evolution. This observation aligns with SVR’s suboptimal predictive performance, validating its limited applicability for this task.

**Figure 9:**
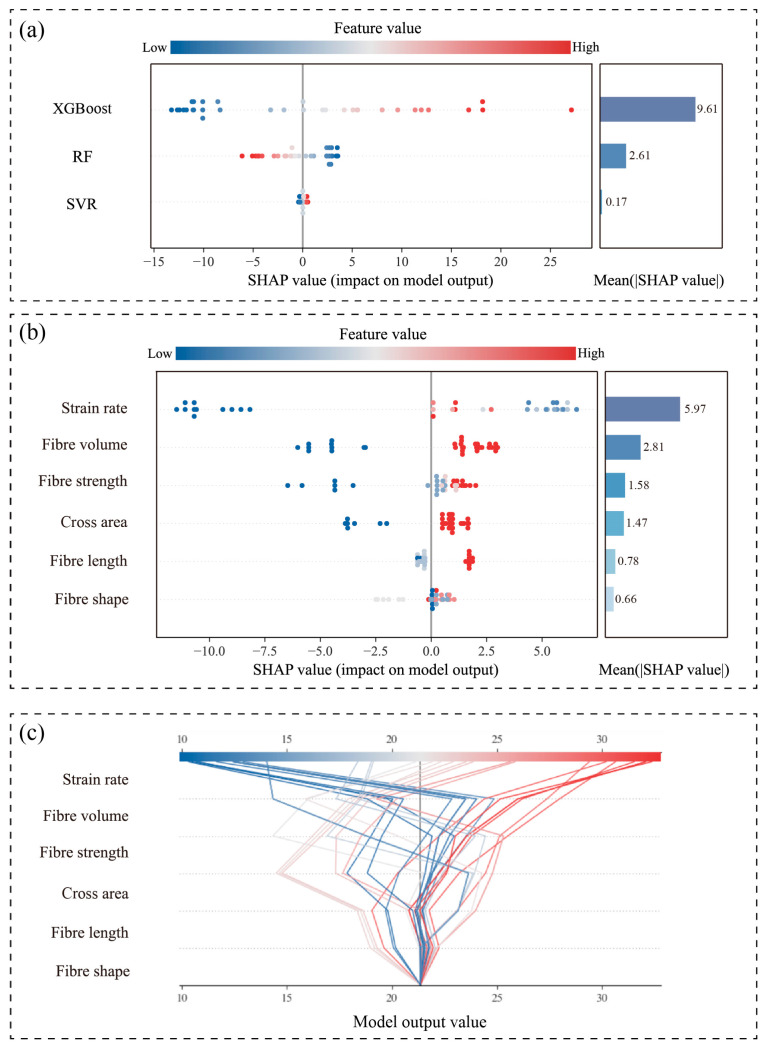
Interpretability results of the FWL model: (**a**) SHAP-based weight contribution of base learners; (**b**) combined SHAP beeswarm and bar plots for FWL output; (**c**) SHAP decision plot.

Figure 9 further elucidates the fusion logic and weighting mechanism of the FWL model. As illustrated in Figure 9a, XGBoost exhibits the highest weighted contribution (mean SHAP value = 9.61) within the meta-model, significantly surpassing those of RF and SVR. This confirms its dominant role in the FWL framework. Figure 9b demonstrates that strain rate and fiber volume exert substantial positive influences across most samples. Higher values (color-coded red) consistently correlate with larger positive SHAP values, which critically elevate the final predictions. This alignment between physical mechanisms and modeling outcomes reflects the inertia-driven delay in crack propagation under increased loading rates, thereby enhancing fracture resistance. Figure 9c displays SHAP decision plots for the FWL model, tracing how cumulative feature contributions progressively refine predictions from baseline values. For high-strength samples (fracture strength > 25 MPa), strain rate and fiber volume deliver the primary positive gains, with concentrated decision paths indicating robust consistency. In contrast, predictions within the low-to-medium strength range exhibit greater dispersion, which reflects the ability of the model to flexibly adjust its weighting mechanism in response to varying input features and to adapt to complex and heterogeneous data patterns.

#### 5.2.2. Local Interpretability Under Varying Strain Rates

**Figure 10:**
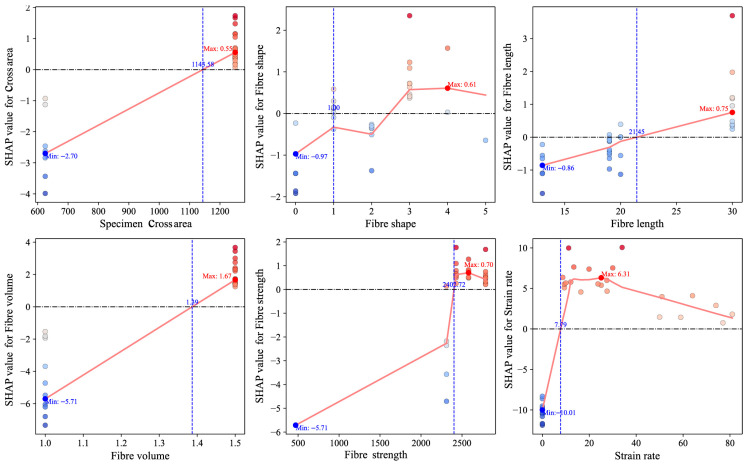
SHAP dependence plots of input features in the FWL model.

To elucidate the predictive response mechanisms of the FWL model under varying input conditions, this study performed interpretability analyses using SHAP dependence plots and localized explanation techniques. As illustrated in Figure 10, most input features exhibit pronounced nonlinear effects on model predictions. Among them, strain rate stands out as the most influential variable, displaying a distinctly nonlinear, piecewise response pattern. As the strain rate increases from 0 to approximately 8 s^−1^, SHAP values shift sharply from strongly negative to positive, indicating a transition from suppressed fracture strength at very low loading rates to rapid enhancement beyond a critical threshold. In the intermediate range of roughly 8–30 s^−1^, SHAP values remain consistently elevated with minor fluctuations, suggesting a sustained positive contribution of strain rate to strength prediction within this sensitivity-dominant interval. Beyond 30 s^−1^, a gradual decline in SHAP values is observed, indicating diminishing marginal contributions at extreme strain rates. This trend likely reflects the onset of microstructural saturation or damage accumulation mechanisms, which limit further gains in predicted fracture strength under high-rate dynamic loading.

Other variables (fiber volume fraction, cross-sectional area, fiber tensile strength) also manifest nonlinear contributions: fiber volume fraction shows linearly increasing SHAP values with higher volume fractions, yet its slope markedly decreases in high-value regions, implying a threshold effect in reinforcement efficacy. Cross-sectional area transitions to positive marginal contributions when exceeding ~1145 mm^2^, reflecting greater strength enhancement in larger specimens. Fiber shape and fiber length exhibit dispersed SHAP patterns with directionality dependent on specific values, revealing complex sample-dependent mechanisms.

**Figure 11:**
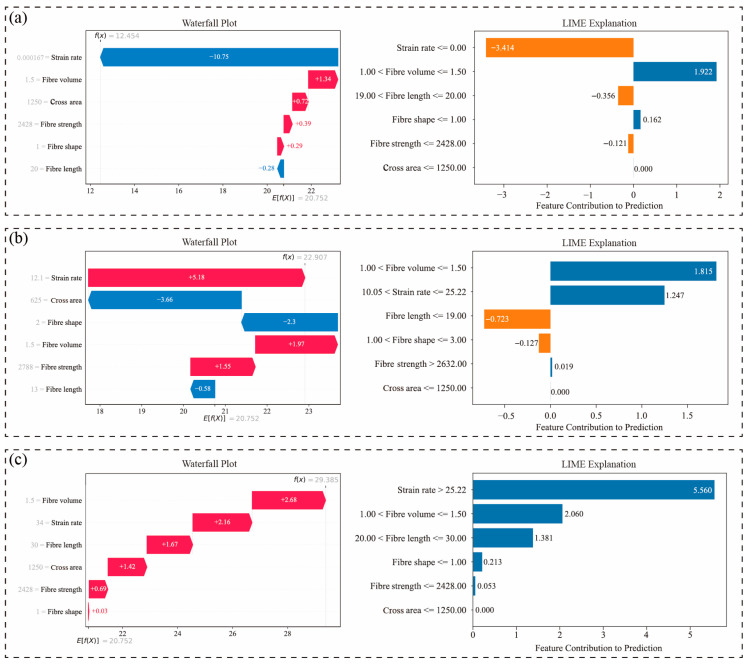
Local explanations of representative samples under varying strain rates based on SHAP and LIME (**a**) low strain rate (0.00167 s^−1^); (**b**) medium strain rate (12.1 s^−1^); (**c**) high strain rate (34 s^−1^).

To further investigate the model’s response mechanisms across distinct strain rate regimes, representative samples from low (0.00167 s^−1^)-, medium (12.1 s^−1^)-, and high (34 s^−1^)-strain-rate intervals were selected. Their SHAP waterfall plots and LIME bar interpretation diagrams are presented in Figure 11, enabling comprehensive analysis of marginal contributions of input variables in individual prediction tasks. Figure 11a illustrates the prediction pathway for the sample at 0.00167 s^−1^. The SHAP waterfall plot reveals that strain rate contributes dominantly as a negative factor (SHAP = −10.75)—the sole significant negative contributor among all variables—driving substantial downward adjustment of the predicted value. This reflects the suppressive effect of low loading rates on fracture strength. LIME analysis further confirms this negative contribution (−3.414), the highest magnitude globally. Concurrently, fiber volume (+1.34) and cross-sectional area (+0.77) emerge as primary positive factors in SHAP analysis, partially offsetting the negative gain from strain rate. This indicates that microstructural characteristics retain limited strengthening capacity under low loading rates, though significantly constrained by strain-controlled suppression. Figure 11b displays results for the medium-strain-rate sample (12.1 s^−1^). Here, strain rate transitions to a strong positive contributor (+5.18), becoming the principal factor elevating predictions. Conversely, cross-sectional area (−3.66) and fiber shape (−2.3) exhibit notable negative contributions, indicating that while loading rate enhances strength in this regime, adverse effects of structural dimensions and morphology persist. LIME results corroborate strain rate and fiber volume as positive drivers, demonstrating high consistency between methods and validating FWL’s interpretative stability under moderate conditions. Figure 11c analyzes the high-strain-rate sample (34 s^−1^). Strain rate remains a primary positive contributor (SHAP = +2.16), though reduced compared to medium rates, suggesting a saturation of strengthening effects under extreme loading. Meanwhile, fiber volume (+2.68) and fiber strength (+1.42) become core positive drivers, indicating that predictions are increasingly governed by fiber properties rather than loading rate alone. LIME confirms the decisive role of strain rate (+5.56, highest among all variables) and highlights increased contributions from fiber volume and fiber length, reflecting enhanced model reliance on structural variables in high-rate regimes.

Collectively, the results reveal distinct response mechanisms of the FWL model to input variables across different strain rate intervals: In the low-strain-rate regime, predictions are primarily influenced by the pronounced negative suppression of strain rate. At intermediate strain rates, fracture strength exhibits a rapid upward trend, accompanied by a significantly enhanced response weight assigned to strain rate by the model. In the high-strain-rate regime, structural variables such as fiber volume fraction and fiber strength become dominant drivers, indicating the model’s transition from loading-dominated to material-property-dominated behavior. These findings imply that the coupling between strain rate and fiber volume fraction plays a critical role in fracture strength prediction. Therefore, optimization of fiber reinforcement dosage should be aligned with the expected loading conditions, as its effectiveness varies significantly across different strain rate regimes.

## 6. Conclusions

This study addresses the prediction of dynamic fracture strength in High-Performance Fiber-Reinforced Concrete (HPFRC) under high strain rates by developing a regression framework integrating feature selection, ensemble modeling, and interpretability analysis. The feature-weighted linear (FWL) ensemble learning method was proposed, systematically evaluating the adaptability and predictive performance of multiple machine learning models across strain rate conditions, with internal model mechanisms analyzed using SHAP and LIME. The primary findings are summarized as follows:

(1) By introducing a feature-driven dynamic weighting mechanism, the FWL model achieves an optimal combination of diverse base learners. Compared with individual models and conventional ensemble methods (e.g., Voting), the FWL model exhibits superior predictive performance on the test set, achieving a high coefficient of determination (R^2^ = 0.908), low prediction errors (RMSE = 2.632, MAE = 1.761), and minimal systematic bias (Bias = –0.577). This confirms its enhanced generalization and robustness under heterogeneous input conditions, outperforming state-of-the-art approaches in capturing high-strain-rate nonlinearity.
(2) Global and local feature response mechanisms, analyzed via SHAP, highlight strain rate and fiber volume fraction as consistently dominant predictors across all models. Their critical coupling effect on fracture strength underscores the interplay between loading rate and material microstructure. Feature selection via correlation analysis and error-driven strategies further validates that these variables effectively mitigate redundancy (e.g., excluding mortar compressive strength and fiber diameter), enhancing model efficiency.
(3) Local interpretability analysis reveals that strain rate exerts a pronounced nonlinear and stage-dependent effect on the predicted fracture strength of HPFRC. Within the low-strain-rate regime (0–8 s^−1^), SHAP values shift from negative to positive, indicating a transition from strength suppression to enhancement as loading rate increases. Within the intermediate-strain-rate regime (8–30 s^−1^), strain rate continues to make a stable and significant positive contribution to strength prediction, suggesting that this interval corresponds to the most prominent phase of dynamic strengthening. Once the strain rate exceeds 30 s^−1^, SHAP values gradually decline, while the importance of structural features, such as fiber volume fraction and tensile strength, increases, indicating a shift in the governing mechanism from loading-rate sensitivity to material-property dominance.

Although the proposed feature-weighted linear (FWL) model demonstrates strong performance in terms of predictive accuracy and interpretability, it currently lacks mechanisms for uncertainty quantification and physics-informed constraints. Future research will aim to incorporate these elements to further enhance the robustness of the model and extend its applicability to a broader range of engineering scenarios involving highly variable loading conditions.

## Figures and Tables

**Table 1:** Description of input and output variables considered in this study.

Category	Variable	Unit	Min	Max	Mean
Input	Mortar compressive strength	MPa	56.00	180.00	152.62
	Specimen cross-sectional area	mm^2^	625.00	1250.00	1110.25
	Fiber diameter	mm	0.20	0.38	0.24
	Fiber shape	-	Hooked, twisted, long smooth, short smooth, Hybrid *		
	Fiber length	mm	13.00	30.00	21.28
	Fiber volume	%	1.00	1.50	1.35
	Fiber tensile strength	MPa	470.00	2788.00	2472.50
	Strain rate	s^−1^	0.000167	100.00	18.97
Output	Fracture strength	MPa	6.00	45.00	20.66

* Fiber shape is a categorical variable such that specific values represent different morphological types.

## Data Availability

The original contributions presented in this study are included in the article. Further inquiries can be directed to the corresponding author.
